# The Rise of Mitochondria in Peripheral Arterial Disease Physiopathology: Experimental and Clinical Data

**DOI:** 10.3390/jcm8122125

**Published:** 2019-12-02

**Authors:** Mégane Pizzimenti, Marianne Riou, Anne-Laure Charles, Samy Talha, Alain Meyer, Emmanuel Andres, Nabil Chakfé, Anne Lejay, Bernard Geny

**Affiliations:** 1 Unistra, Translational Medicine Federation of Strasbourg (FMTS), Faculty of Medicine, Team 3072 «Mitochondria, Oxidative Stress and Muscle Protection», 11 Rue Humann, 67000 Strasbourg, France; megane.pizzimenti@hotmail.fr (M.P.); marianne.riou@chru-strasbourg.fr (M.R.); anne.laure.charles@outlook.fr (A.-L.C.); samy.talha@chru-strasbourg.fr (S.T.); alain.meyer1@chru-strasbourg.fr (A.M.); anne.lejay@chru-strasbourg.fr (A.L.); 2 Physiology and Functional Exploration Service, University Hospital of Strasbourg, 1 Place de l’Hôpital, 67091 Strasbourg CEDEX, France; 3 Internal Medicine, Diabete and Metabolic Diseases Service, University Hospital of Strasbourg, 1 Place de l’Hôpital, 67091 Strasbourg CEDEX, France; emmanuel.andres@chru-strasbourg.fr; 4 Vascular Surgery and Kidney Transplantation Service, University Hospital of Strasbourg, 1 Place de l’Hôpital, 67091 Strasbourg CEDEX, France

**Keywords:** peripheral arterial disease, ischemia-reperfusion, mitochondria, oxidative stress, reactive oxygen species, antioxidant, calcium retention capacity, apoptosis

## Abstract

Peripheral arterial disease (PAD) is a frequent and serious condition, potentially life-threatening and leading to lower-limb amputation. Its pathophysiology is generally related to ischemia-reperfusion cycles, secondary to reduction or interruption of the arterial blood flow followed by reperfusion episodes that are necessary but also—per se—deleterious. Skeletal muscles alterations significantly participate in PAD injuries, and interestingly, muscle mitochondrial dysfunctions have been demonstrated to be key events and to have a prognosis value. Decreased oxidative capacity due to mitochondrial respiratory chain impairment is associated with increased release of reactive oxygen species and reduction of calcium retention capacity leading thus to enhanced apoptosis. Therefore, targeting mitochondria might be a promising therapeutic approach in PAD.

## 1. Introduction

Peripheral arterial diseases (PAD) is a major concern for public healthcare, affecting more than 200 million individual worldwide [1,2]. Its prevalence varies from 3% to 10%, but can reach up to 20% in the elderly population [3].

PAD is defined by a narrowing of the peripheral arterial vasculature. It mostly affects lower limbs, leading to overall functional disability and reduced quality of life. Initially asymptomatic, PAD progressively compromises lower limb vascularization, leading to obstruction of the vessels by atheroma. PAD includes all stages of the disease, from asymptomatic with abolition of distal pulses, to intermittent claudication or critical limb threatening ischemia (CLTI) characterized by rest pain and/or ulcers. PAD is also often associated with cognitive dysfunction characterized by reduced performance in nonverbal reasoning, reduced verbal fluency, and decreased information processing speed [4]. Thus, PAD is a serious condition threatening both limb (risk of amputation) and vital prognosis of the patients. Indeed, despite recent therapeutic progress, morbidity and mortality rates remain incompressible around 20% and 15% five years after a diagnosis of symptomatic or asymptomatic PAD [5]. On average, the life expectancy of claudicating patients is reduced by 10 years, with a majority of death attributable to cardiovascular causes. 

The treatment of PAD is mainly based on revascularization of the ischemic limb [6]. Nevertheless, this surgical procedure is not always possible, notably when the vascular state is too precarious or when the local evolution is too advanced. It appears therefore important to better understand PAD pathophysiology to offer optimal patient care. Indeed, insufficient oxygen supply was long presumed to be the main and sole cause for PAD symptoms. However, recent advances in understanding PAD physiopathology identified mitochondria as a key element in the deleterious process of PAD [7,8].

**Figure 1:**
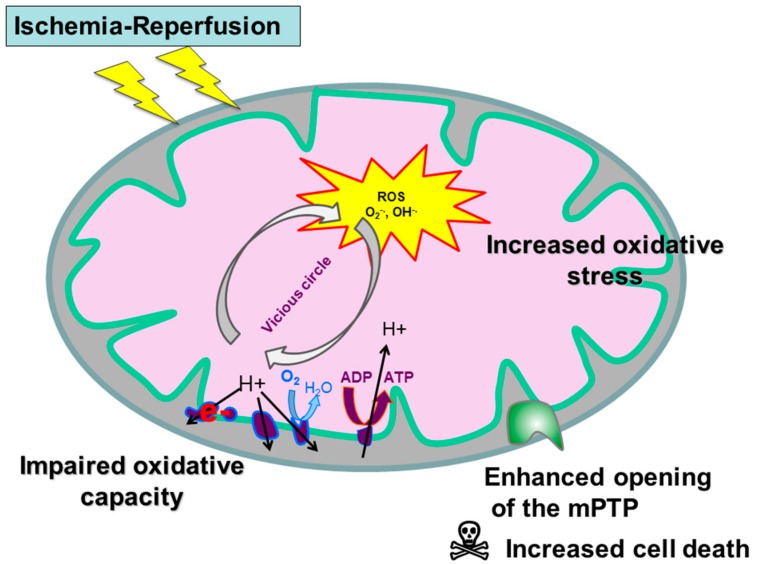
Mitochondrial dysfunction during peripheral arterial disease (PAD). mPTP: mitochondrial permeability transition pore.

Thus, skeletal muscles alterations significantly participate in PAD injuries, modulating its prognosis, and this review aims to describe muscle mitochondrial dysfunctions. Particularly, we will analyze data focused on mitochondrial respiratory chain respiration, on reactive oxygen species release and on mitochondrial calcium retention capacity which decrease is associated with enhanced apoptosis (Figure 1). 

## 2. Mitochondrial Function under Normal and Pathological Conditions

### 2.1. Normal Condition

Life requires energy, and this energy is stored in adenosine triphosphate (ATP) molecules, that are produced in the mitochondria by oxidative phosphorylation and assessed through mitochondrial respiration determination. Specifically, the oxidation of nutrients through the Krebs cycle provides reduced coenzymes (the reduced form of nicotinamide adenine dinucleotide (NADH)) and to a lesser extent flavin adenine dinucleotide (FADH_2_), which are electron donors. This flow of electrons is supported by different redox reactions provided by the four complexes of the mitochondrial respiratory chain, up to the reduction of molecular oxygen in water. The respiratory complexes use the energy generated by this electron transfer to allow an active translocation of protons from the matrix to the inter-membrane mitochondrial space. This expulsion of protons results in the generation of a concentration gradient and a mitochondrial membrane potential across the inner membrane. ATP synthases use the transmembrane protonmotive force as a source of energy to drive a mechanical rotary mechanism leading to the chemical synthesis of ATP from ADP and Pi.The F_1_F_0_ ATP synthase enzymes allow proton flux and ATP synthesis through a and c subunits of the F_0_ domain. The maintenance of this electrochemical gradient, also called protomotive force, is an essential element for the energetic role of the mitochondria [9,10,11].

Mitochondrial respiration generates free radicals derived from oxygen, the reactive oxygen species (ROS). A free radical is a chemical species containing an unpaired electron. Extremely unstable, this compound can react with more stable molecules to match its electron. It can then pull out an electron and behave as an oxidant, usually leading to the formation of new radicals in the chain and causing significant cell damage. Main ROS comprise superoxide anion, the hydroxyl radical and the highly reactive compound hydrogen peroxide (H_2_O_2_). Detoxification systems exist, enzymatic (superoxide dismutase, catalase, glutathione peroxidase) or not (vitamins and trace elements). 

Nevertheless, when the radical production remains contained below a certain threshold, the ROS activate defenses pathways involving the development of cellular antioxidants and mitochondrial biogenesis. Also named mitohormesis, these mechanisms constitute one of the therapeutic targets that can limit the lesions linked to repeated cycles of ischemia-reperfusion in PAD [12].

### 2.2. Ischemic Condition

During ischemia, ATP is generated by anaerobic glycolysis, leading to glycogen storage depletion, anaerobic metabolism activation and local lactic acidosis. The resulting depletion of ATP reduces the function of membrane pumps and causes cellular edema. Indeed, the cell tends to correct the acidosis by expelling the H+ ions via the Na+/H+ exchanger, thus saturating the cytoplasm with Na + ions and causing an osmotic shift to the cytoplasm. Cell edema is aggravated by Na+/K+ ATP-dependent exchanger dysfunction due to lack of ATP, which also leads to Na+ accumulation in the cytoplasm. Acidosis also activates mediators, such as phospholipaseA2, that metabolize membrane phospholipids to arachidonic acid, a precursor of inflammatory mediators such as leukotrienes and prostaglandins. Ischemia will also initiate conversion of xanthine dehydrogenase to xanthine oxidase [13].

Reperfusion is able to prevent the irreversible damages of ischemia. Nevertheless, this process also generates lesions that aggravate the pre-existing tissue damages. At the cellular level, reoxygenation interrupts the lesions induced by ischemia, but causes reperfusion injury. During the first few minutes of reperfusion, the rapid correction of acidosis increases the cytosolic Ca^2+^, thus promoting the opening of the mitochondrial permeability transition pore [14]. This opening causes a sudden change in the mitochondrial membrane permeability, resulting in energy collapse incompatible with cell survival and inducing the release of pro-apoptotic factors from the inter-membrane mitochondrial space to the cytosol, leading to cell death. It is the intrinsic mitochondrial apoptosis pathway.

In parallel, reperfusion generates massive oxidative stress since xanthine oxidase and succinate, produced during ischemia, catalyzes the formation of uric acid from hypoxanthine and of ubiquinol, respectively, accompanied by the formation of large amounts of free radicals. Very interestingly, Chouchani et al. demonstrated a conserved metabolic response of tissues to ischemia-reperfusion (IR) revealing that reducing ischemia-induced succinate increase and its oxidation after reperfusion might be an important therapeutic during IR settings. [15,16,17]. 

The ROS thus produced exceed the cellular antioxidant defenses creating a vicious circle. Production of free radicals will cause a dysfunction of the mitochondrial respiratory chain, which in turn generate more ROS. Such ROS overproduction leads to several deleterious effects: lipid peroxidation, protein oxidation and DNA mutations, but also to the opening of the mitochondrial permeability transition pore.

## 3. Mitochondrial Oxidative Capacities in PAD

### 3.1. Experimental Data

Impairments in mitochondrial respiration were observed in both claudicating and CLTI experimental models. Indeed, significant reduction in mitochondrial complexes I, II and IV activities was found in muscles of rats submitted to hindlimb ischemia-reperfusion compared to contralateral muscles [18,19,20,21]. Similarly, mouse models of CLTI presented decreased activities of the complexes I, III and IV in ischemic muscles compared with controls [22].

The respiratory impairments were shown to be strain-, muscle-, age- and disease- specific. Indeed, mitochondrial respiration was affected by hindlimb ischemia-reperfusion in limb muscles of BALB/c mice, but not of C57BL/6 mice [23]. Secondly, ischemia-reperfusion injury was shown to affect more severely the respiration in glycolytic muscles than in oxidative ones [24]. Furthermore, the impairments in mitochondrial respiration observed in young mice were greater in older animals submitted to ischemia-reperfusion injury [25]. Lastly, the decline in mitochondrial oxidative capacity was more severe in diabetic rats compared to non-diabetic animals (Table 1) [26].

### 3.2. Clinical Data

Oxygraphy measurements in PAD patients revealed significantly altered respiratory activity, notably of complexes I, III and IV, and of the acceptor control ratio [27,28,29,30]. Interestingly, more recent studies reported no difference in the mitochondrial respiration rate between PAD patients and healthy controls, despite alterations in O_2_ delivery, tissue-reoxygenation and ATP synthesis rate during exercise [31,32]. These conflicting findings could be explained by disparities in disease severity. Indeed, the alterations in mitochondrial oxidative capacity may have been the result of factors associated with higher morbidity in PAD, such as sarcopenia [33,34,35].

Mitochondrial energy metabolism was shown to decrease in PAD patients compared to controls [36,37]. In contrast, Hou et al. found similar mitochondrial ATP production rate in both PAD patients and healthy controls [38]. Again, such differences might be explained by differences in disease severity or morbidity factors rate. 

It is important to note that patients suffering from both PAD and type II diabetes (DT2) are more susceptible to reduced O2 consumption and mitochondrial oxidative phosphorylation compared to patients with PAD alone or to controls (Table 2) [39,40].

## 4. Reactive Oxygen Species Production, Proteins, Lipids and DNA Alterations and Impaired Antioxidant Defense, in PAD

The interaction between mitochondria and oxidative stress in skeletal muscle is modulated by repeated cycles of ischemia-reperfusion in the context of PAD. The vascular damages create an imbalance between oxygen supply and demand during efforts, generating a situation of ischemia; followed by a situation of reperfusion when the patient is at rest. Repetition of ischemia and reperfusion cycles are deleterious for skeletal muscle and lead to myopathy and to remote organ damage [7,28,41].

### 4.1. Experimental Data

ROS production was found increased in both animal models of PAD (acute ischemia-reperfusion and CLTI) as compared to controls, using either measurements of 1) free radical species by electron paramagnetic resonance spectroscopy, 2) dihydroethidium (DHE) by epifluorescence microscopy or 3) H_2_O_2_ by Amplex Red perioxide assay [24,42,43]. Interestingly, ROS production was greater in PAD animals presenting with hypercholesterolemia or diabetes [26,44]. These evidences suggest an association between PAD comorbidity factors and enhanced mitochondrial dysfunction. 

Furthermore, deleterious effects of oxidative stress were also observed in ischemic skeletal muscles, as highlighted by higher levels of oxidative stress markers (superoxide dismutase [45], protein carbonyls and 4-hydroxy-2-nonenanal protein (HNE) adducts) [22], and elevated DNA alterations [46].

Finally, antioxidant defenses have been shown to be impaired by ischemia-reperfusion. Indeed, alterations in the expression of superoxide dismutase 1 and 2 (SOD1 and SOD2), catalase and manganese superoxide dismutase (MnSOD) were observed in ischemic muscles compared with controls (Table 3) [22,45,47].

### 4.2. Clinical Data

Similar to the findings on experimental models, PAD patients displayed increased mitochondria-derived ROS production characterized by elevated levels of free radical species [32].

Moreover, oxidative damages were also observed in patients suffering from PAD, as reported by higher levels of oxidative stress markers (protein carbonyl groups, HNE-protein adducts and lipid hydroperoxides), and elevated DNA alterations [28,49,50,51,52,53].

Lastly, evidence of reduced antioxidant defenses has been shown in PAD patients, notably with altered activities of the antioxidant enzymes SOD, catalase and glutathione peroxidase (Table 4) [28].

## 5. Mitochondrial Implication in Apoptosis during PAD

### 5.1. Experimental Data

Rodent models of PAD displayed elevated protein expression of the apoptotic factors cleaved-caspase 3, cleaved-poly (ADP-robose) polymerase (PARD) and mitochondrial and cytosolic Bcl2-associated X (Bax), and reduced protein expression of the anti-apoptotic factor Bcl-2, compared with controls [21,54,55]. Furthermore, a decrease in mitochondrial calcium retention capacity was observed in ischemic limbs compared with contralateral ones (Table 5) [25,44,47,56,57].

### 5.2. Clinical Data

Human investigations also showed a clear implication of apoptosis in PAD pathophysiology. Indeed, PAD patients displayed elevated levels of genes mediating apoptosis [58], increased DNA fragmentation and caspase-3 activity [59]. Additionally, multiple studies reported higher levels of apoptotic cells in different cell types, notably endothelial cells and lymphocytes [60,61]. Interestingly, another study reported similar levels of endothelial apoptosis in both PAD and control groups. This result is likely due to disparities in patient’s selection, with patients presenting with lower associated risk factors (Table 6) [62].

## 6. Conclusions

In summary, PAD is a public health issue even when poorly symptomatic [63], and mitochondria are importantly involved in its pathophysiology. Not only because mitochondrial alterations reduce the energy available for cell function, but also because mitochondria participate in the increased ROS production (and therefore to a greater oxidative stress) and in the enhanced opening of the mitochondrial permeability pore which favor the intrinsic pathway of cell apoptosis. These data account for the fact that skeletal muscle mitochondrial function might be considered as a prognostic factor in the setting of PAD in humans. On the other hand, if ROS production remains contained below a certain threshold, mitohormesis and stimulation of the antioxidant defenses can be protective supporting that mitochondrial function modulation might be a therapeutic target. Thus, further studies focused on mitochondria are warranted to optimize the care of patients presenting with PAD.

## Figures and Tables

**Table 1:** Mitochondrial oxidative capacity and peripheral arterial disease in selected experimental studies.

Animals	Study Design Ischemia-Reperfusion Duration	Outcomes Measured	Main Results	Reference
Mice, n = 25 young (23 ± 1 weeks) and old (84 ± 1 weeks)	Unilateral tourniquet I: 2 h/R: 2 h	Skeletal muscle mitochondrial capacity (by oxygraphy)	Impaired mitochondrial respiration in young PAD mice compared with sham (V_ADP_ 33.0 ± 2.4 for the contralateral limb versus 18.4 ± 3.8 for the ischemic limb, *p* < 0.01). Enhanced impairment in old PAD mice (V_ADP_ 5.9 ± 2.7 pmol/s/mg wet weight, *p* < 0.001).	Paradis et al., 2019, Antioxidants [25].
Rats, n = 36 diabetic and non-diabetic	Aortic banding I: 3 h/R: 2 h	Skeletal muscle mitochondrial capacity (by oxygraphy)	Significant decline in mitochondrial respiration after ischemia-reperfusion injury in diabetic rats compared to non-diabetic (*p* < 0.05).	Pottecher et al., 2018, Front Physiol [26].
Mice, n = 69 BALB/c (ischemia susceptible) and C57BL/6 (ischemia protected)	Aortic banding I and R: duration not specified	Skeletal muscle mitochondrial capacity (by western blot and oxygraphy)	Skeletal muscle mitochondrial impairments in BALB/c limb muscle but not in C57BL/6 (*p* < 0.01).	Schmidt et al., 2017, J Vasc Surg [23].
Mice, n = 22	Aortic banding I: 3 h/R: 2 h	Skeletal muscle mitochondrial capacity (by oxygraphy)	Decreased mitochondrial respiration in glycolytic versus oxidative muscles.	Charles et al., 2017, Front Physiol [24].
Rats, n = 12 Old 71-73 weeks	Unilateral tourniquet I: 3 h/R: 2 h	Skeletal muscle mitochondrial capacity (by oxygraphy)	Reduced mitochondrial complexes I, II and IV activities in PAD muscles compared with contralateral ones (V_MAX_ 7.34 ± 1.5 versus 2.87 ± 1.22 µmol O_2_/min/g dry weight for PAD muscles, *p* < 0.05).	Pottecher et al., 2016, Fundam Clin Pharmacol [18].
Rats n = 28	Aortic banding I: 3 h/R: 2 h	Skeletal muscle mitochondrial capacity (by oxygraphy)	IR reduced V(max) (−21.2%, 6.6 ± 1 versus 5.2 ± 1 μmol O_2_/min/g dry weight, *p* = 0.001), V(succ) (−22.2%, *p* = 0.032), and V(TMPD) (−22.4%, *p* = 0.033).	Mansour et al., 2012, J Vasc Surg [21].
Rats, n = 22	Unilateral tourniquet I: 5 h R: 5 min	Skeletal muscle mitochondrial capacity (by oxygraphy)	Reduced mitochondrial complexes I, II and IV activities in PAD rats compared with sham (V_MAX_ 4.4 ± 0.4 versus 8.7 ± 0.5 µmol O_2_/min/g dry weight, *p* < 0.001).	Thaveau et al., 2010, Fundam Clin Pharmacol [19].
Mice, n = 48	CLTI Sequential left femoral and iliac ligations R: week 12	Skeletal muscle mitochondrial capacity (by oxygraphy)	Reduced activity of complexes I (by 34%), III (by 45%) and IV (by 42%) in ischemic muscles compared with controls (*p* < 0.05).	Pipinos et al., 2008, Am J Physiol Regul Integr Comp Physiol [22].
Rats, n = 20	Unilateral tourniquet I: 5 h	Respiration of isolated mitochondria (by polarographic analysis)	Inhibition of the mitochondrial respiratory chain	Brandão et al., J Surg Res. 2003 [20].

CLTI: critical limb threatening ischemia; I: ischemia; PAD: peripheral arterial disease; R: reperfusion. TMPD, N, N, N′, N′-tetramethyl-p-phenylenediamine dihydrochloride.

**Table 2:** Mitochondrial oxidative capacities and peripheral arterial disease in clinical studies.

Population	Number Studied (Symptomatic/Controls)	Outcomes Measured	Main Results	Reference
Early stage PAD	10/11	O_2_ delivery, tissue oxygenation and Vmax (by high-resolution respirometry). Skeletal muscle mitochondrial capacity (by oxygraphy)	PAD patients exhibited significantly lower O_2_ delivery (*p* < 0.05), tissue-reoxygenation (58 ± 3 % for controls versus 44 ± 3 % for PAD patients, *p* < 0.05) and Vmax (*p* < 0.05) during exercise, compared with healthy controls. No differences were found in the mitochondrial respiration rate.	Hart et al., 2018, Am J Physiol Heart Circ Physiol [31].
Claudicant PAD	10/12	Skeletal muscle mitochondrial capacity (by oxygraphy)	No differences were found in the mitochondrial respiration rate between PAD patients and healthy controls.	Hart et al., 2018, Exp Physiol [32].
Claudicant PAD; claudicant PAD + DT2	15 (PAD)/15 (PAD + DT2)/10 (controls)	Skeletal muscle mitochondrial capacity (by oxygraphy)	Significant reduction in oxygen consumption in the PAD+DT2 group compared with the PAD group or the control group (*p* < 0.05). No differences were found in the mitochondrial respiration rate between PAD patients and healthy controls.	Lindegaard et al., 2017, Int Angiol [39].
Patients with low ABI	82 (ABI of 0.90 to 1.10)/281 (ABI of 1.11 to 1.40)	Phosphocreatine recovery (by phosphorus-31 magnetic resonance spectroscopy)	Significantly lower muscle mitochondrial energy production in patients with lower ABI, compared with those with higher ABI (20.8 ms^−1^ for higher ABI versus 19.3 ms^−1^ for lower ABI, *p* = 0.015).	AlGhatrif et al., 2017, J Am Heart Assoc [36].
PAD (no stage specified)	30/30	Skeletal muscle mitochondrial capacity (by oxygraphy)	PAD subjects presented significantly lower respiratory activity compared with controls (*p* < 0.05).	Koutakis et al., 2015, J Histochem Cytochem [27].
Claudicant PAD + neuropathy + DT2	7/14	Phosphocreatine recovery (by phosphorus-31 magnetic resonance spectroscopy)	Reduced mitochondrial oxidative phosphorylation in DT2 patients with lower extremity complications (neuropathy and PAD) (*p* < 0.05).	Tecilazich et al., 2013, J Vasc Surg [40].
Claudicant PAD; CLI	25/16	Skeletal muscle mitochondrial capacity (by spectrophotometry)	Decreased activity of complexes I, III and IV in PAD muscle compared to control (*p* < 0.05).	Pipinos et al., 2006, Free Radic Biol Med [28].
Claudicant PAD; CLI	9/9	Skeletal muscle mitochondrial capacity (by oxygraphy)	Significantly lower respiratory rates, and lower acceptor control ratio (2.90 ± 0.20 for controls versus 1.41 ± 0.10 for PAD) in patients with PAD compared with controls (*p* < 0.05).	Pipinos et al., 2003, J Vasc Surg [29].
Claudicant PAD	7/11	ATP synthesis (by luminometer)	Similar mitochondrial ATP production rate were in PAD patients and healthy controls.	Hou et al., 2002, Clin Physiol Funct Imaging [38].
Claudicant PAD	17/9	Skeletal muscle mitochondrial capacity (by spectrophotometry)	Significant reduction in NADH dehydrogenase and ubiquinol-cytochrome c oxidoreductase activity by 27% and 38%, respectively, in PAD compared with controls (*p* < 0.05).	Brass et al., 2001, Am J Physiol Heart Circ Physiol [30].
Claudicant PAD	12/14	Phosphocreatine and ADP recovery (by phosphorus-31 magnetic resonance spectroscopy)	Defective phosphocreatine (44 ± 3 s for controls versus 137 ± 41 s for PAD) and ADP recovery (29 ± 2 s versus 60 ± 10 s for PAD) in PAD compared with controls (*p* < 0.05).	Pipinos et al., 2000, J Vasc Surg [37].

ABI: ankle brachial index; CLTI: critical limb threatening ischemia; DT2: type II diabetes; PAD: peripheral arterial disease.

**Table 3:** Reactive oxygen species production during peripheral arterial disease in experimental studies.

Animals	Study Design Ischemia-Reperfusion Duration	Outcomes Measured	Main Results	Reference
Mice, n = 7 ApoE-/- versus ApoE+/+	CLTI Sequential right femoral and iliac ligations. R: day 40	Free radical measurement (by electron paramagnetic resonance spectroscopy)	Enhanced ROS production in muscles of ApoE-/- (+63.6%) and ApoE+/+ (+41.4%) mice compared with contralateral muscles.	Lejay et al., 2019, Eur J Vasc Endovasc Surg [44].
Rats, n = 36 diabetic versus non-diabetic	Aortic banding I: 3 h/R: 2 h	DHE measures of ROS (by epifluorescence microscopy)	Increase in normalized DHE fluorescence in diabetic PAD compared to diabetic controls (*p* < 0.001).	Pottecher et al., 2018, Front Physiol [26].
Mice, n = 20	CLTI Left femoral ligation. R: day 21	mtDNA damage quantification (by quantitative PCR)	Increase in mtDNA damages in ischemic muscles of PAD mice compared with sham (*p* < 0.05).	Miura et al., 2017, Int J Mol Sci [46].
Mice, n = 20	CLTI Sequential right femoral and iliac ligations. R: day 30	Antioxidant quantification (by quantitative PCR)	Significant decrease in mRNA expression of the antioxidant enzymes SOD1 (0.39 ± 0.10 for sham limb versus 0.10 ± 0.06 for ischemic limb), SOD2 (0.32 ± 0.16 versus 0.11 ± 0.07) and catalase (0.38 ± 0.04 versus 0.22 ± 0.11) in ischemic muscles compared with control ones (*p* < 0.05).	Lejay et al., 2017, Front Physiol [47].
Mice, n = 22	Aortic banding I: 3 h/R: 2 h	Free radical measurement (by electron paramagnetic resonance spectroscopy)	Ischemia-reperfusion injury increased ROS production in ischemic muscles compared to no ischemic contralateral (+79.15 ± 28.72%, *p* = 0.04).	Charles et al., 2017, Front Physiol [24].
Mice, n = 6	CLTI Right femoral ligation. R: day 10	H_2_O_2_ measurement (by Amplex Red assay)	Significant increase in H_2_O_2_ level in ischemic muscles compared with sham ones (*p* < 0.05).	Kwon et al., 2016, Int J Pharm [43].
Mice, n = 28	CLTI Sequential right femoral and iliac ligations. R: day 30.	Free radical measurement (by electron paramagnetic resonance spectroscopy) DHE measures of ROS (by epifluorescence microscopy)	CLI induced a significant increase in ROS production in ischemic muscles compared with controls. DHE staining was higher in ischemic muscles (*p* < 0.01).	Lejay et al., 2015, Eur J Vasc Endovasc Surg [42].
Rats, n = 35	Aortic banding I: 2 h/R: 10 min and 2 h	DHE staining (by epifluorescence microscopy)	ROS increased significantly after ischemia alone (+324 ± 66%, *p* = 0.038), normalized after 10 min of reperfusion, and increased again at 2 h of reperfusion (+349.2 ± 67%, *p* = 0.024). Oxidative stress preceded skeletal muscle mitochondrial dysfunction.	Guillot et al., 2014, J Vasc Surg [48].
Mice, n = 18	Unilateral tourniquet I: 3 h/R: 4 h	Superoxide anion production measurement (by luminometer); Quantification of MnSOD (by Western blot)	Increased superoxide production and decreased activity of the mitochondria-targeted SOD isoform) in the ischemia-reperfusion group.	Tran et al., 2011, Eur J Pharmacol [45].
Mice, n = 48	CLTI Sequential left femoral and iliac ligations R: week 12	Protein carbonyls, HNE adducts and MnSOD expression quantification (by reverse phase protein lysate microarray)	Significantly higher expression of protein carbonyls, HNE adducts and MnSOD in ischemic muscles compared with controls (*p* < 0.05).	Pipinos et al., 2008, Am J Physiol Regul Integr Comp Physiol [22].

CLTI: critical limb threatening ischemia; DHE: dihydroethidium; dw: dry weight; HNE: 4-hydroxy-2-nonenal; I: ischemia; MnSOD: manganese superoxide dismutase; mtDNA: mitonchondrial DNA; PAD: peripheral arterial disease; PCR: polymerase chain reaction; R: reperfusion; ROS: reactive oxygen species; SOD: superoxide dismutase.

**Table 4:** Reactive oxygen species production during peripheral arterial disease in clinical studies.

Population	Number Studied (Symptomatic/Controls)	Outcomes Measured	Main Results	Reference
Claudicant PAD	10/34	Mitochondrial DNA copy number (by quantitative PCR)	Significant association between disease severity and increased mitochondrial DNA copy number (*p* < 0.05).	McDermott et al., 2018, Vasc Med [49].
Claudicant PAD	10/12	Free radical measurement (by electron paramagnetic resonance spectroscopy)	Significant increase in mitochondria-derived ROS production in PAD (1.0 ± 0.36 AU/mg tissue for controls versus 4.3 ± 1.0 AU/mg tissue for PAD, *p* < 0.05).	Hart et al., 2018, Exp Physiol [32].
Claudicant PAD; CLTI	28 claudicants/25 CLTI/25 controls	Carbonyl groups quantification (by quantitative fluorescence microscopy)	Observation of a 25% increase in carbonyl groups (markers of oxidative damage) in myofibers of all PAD patients compared with controls (*p* < 0.05).	Koutakis et al., 2014, Redox Biol [50].
Claudicant PAD; CLTI	34/21	Carbonyl groups and HNE adducts quantification (by quantitative fluorescence microscopy)	Significant increase in carbonyl groups (30%, *p* < 0.0001) and HNE adducts (40%, *p* < 0.0001) in PAD myofibers compared to controls.	Weiss et al., 2013, J Transl Med [51].
Claudicant PAD; CLTI	16/10	Lipid hydroperoxides measurement (by ferrous oxidation/xylenol orange technique); Protein carbonyls measurments (using an Enzyme Immuno-Assay kit); HNE detection (by western blot); Antioxidant activity (by spectrophotometry)	Higher levels of lipid hydroperoxides (12.45 ± 0.74 mmol/g wet weight for controls versus 20.32 ± 1.02 for PAD), protein carbonyls (0.22 ± 0.02 nmol/mg for controls versus 0.35 ± 0.04) and HNE (191.2 ± 7.17 total binding versus 226.4 ± 10.4) was found in PAD patients compared to controls (*p* < 0.05). Significant decrease in SOD activity, and increase in catalase and glutathione peroxidase activities.	Pipinos et al., 2006, Free Radic Biol Med [28].
Claudicant PAD	9 claudicants	Quantification of mitochondrial DNA injury (by PCR)	Substantial injury to mitochondrial DNA in PAD patients occurring bilaterally in patients with unilateral PAD.	Brass et al., 2000, Vasc Med [52].
Claudicant PAD	8/10	Quantification of mitochondrial DNA injury (by PCR)	Accumulation of 4977-bp mitochondrial deletion frequency in patients with PAD compared with controls (0.05 ± 0.01 % for controls versus 0.43 ± 0.28 % for the less-affected limb versus 0.88 ± 0.53 % for the worse-affected limb, *p* < 0.05).	Bhat et al., 1999, Circulation [53].

CLTI: critical limb threatening ischemia; HNE: 4-hydroxy-2-nonenal; mtDNA: mitochondrial DNA; PAD: peripheral arterial disease; PCR: polymerase chain reaction; SOD: superoxide dismutase.

**Table 5:** Mitochondrial implication in apoptosis during peripheral arterial disease in experimental studies.

Animals	Study Design Ischemia-Reperfusion Duration	Outcomes Measured	Main Results	Reference
Mice, n = 7 ApoE-/- versus ApoE+/+	CLTI Sequential right femoral and iliac ligations.	Calcium retention capacity (by spectrofluometry)	Impairment in calcium retention capacity in ischemic muscles of ApoE-/- and ApoE+/+ mice compared with contralateral muscles (*p* = 0.001).	Lejay et al., 2019, Eur J Vasc Endovasc Surg [44].
Mice, n = 25 young (23 weeks) versus aged (84 weeks) C57Bl6J	Unilateral tourniquet I: 2 h/R: 2 h	Calcium retention capacity (by spectrofluometry)	Significant reduction in calcium retention capacity in young (-60.9 ± 7.3%) and aged (-60.9 ± 4.6%) mice compared with sham (*p* < 0.001).	Paradis et al., 2019, Antioxidants [25].
Rats, n = 12	CLTI Left femoral ligation. R: day 14	Protein expression of indicators of apoptosis (by Western blot)	Higher expression of proteins cleaved-caspase 3, cleaved-PARP and mitochondrial Bax in CLTI muscles compared with sham ones (*p* < 0.05).	Hsu et al., 2019, Am J Transl Res [54].
Mice, n = 16	Unilateral tourniquet I: 2 h/R: 2 h	Calcium retention capacity (by spectrofluometry)	Decrease in calcium retention capacity in ischemic limbs compared with contralateral ones (-61.1 ± 6.8%, *p* < 0.01).	Tetsi et al., 2019. Antioxidants [56].
Mice, n = 20	CLTI Sequential right femoral and iliac ligations. R: day 30.	Calcium retention capacity (by spectrofluometry)	Significant reduction of calcium retention capacity in ischemic limbs compared with contralateral ones (*p* < 0.001).	Lejay et al., 2018, Eur J Vasc Endovasc Surg [57].
Mice, n = 20	CLTI Sequential right femoral and iliac ligations.	Calcium retention capacity (by spectrofluometry)	Lower calcium retention capacity in ischemic limbs compared with controls (*p* < 0.001).	Lejay et al., 2017, Front Physiol [47].
Rats, n = 16	CLTI Right femoral ligation. R: day 14	Protein expression of indicators of apoptosis and anti-apoptotic factor (by Western blot)	Higher expression of the proteins cleaved-caspase 3, cleaved-PARP and cytosolic Bax in CLTI muscles compared with sham ones (*p* < 0.001). Lower expression of the anti-apoptotic marker Bcl-2 in CLTI muscles compared with sham ones (*p* < 0.001).	Sheu et al., 2015, J Transl Med [55].
Rats n = 28	Aortic banding I: 3 h/R: 2 h	Quantification of gene expression (by quantitative PCR)	IR increased Bax (63.4%, *p* = 0.020) and Bax/Bcl-2 ratio (+84.6%, *p* = 0.029). SODs and GPx messenger RNA were not modified, but glutathione tended to be decreased after IR.	Mansour et al., 2012, J Vasc Surg [21].

CLTI: critical limb threatening ischemia; I: ischemia; IR: ischemia-reperfusion; PAD: peripheral arterial disease; R: reperfusion.

**Table 6:** Mitochondrial implication in apoptosis during peripheral arterial disease in clinical studies.

Population	Number Studied (Symptomatic/Controls)	Outcomes Measured	Main Results	Reference
Claudicant PAD	130/36	Caspase activity measurement (by caspase Assay)	No difference observed in apoptosis between the PAD and the control group (*p* = 0.463).	Gardner et al., 2014, Angiology [62].
Claudicant PAD	156/16	Caspase activity measurement (by caspase Assay)	Higher percentage of endothelial cell apoptosis in the PAD group compared with the control group (+164%, *p* < 0.001).	Gardner et al., 2014, Int J Vasc Med [60].
Claudicant PAD	19/18	Quantification of gene expression (by quantitative PCR)	Upregulation in genes mediating apoptosis: *BCL-2*, *G0S2*, *KLF6*, *PTP4A1* and *CFLAR*.	Masud et al., 2012, J Clin Bioinforma [58].
Claudicant PAD	10/10	Detection and quantification of apoptosis (by fluorescence microscopy)	Higher percentage of late apoptotic lymphocytes (by 33%) in the PAD patients compared with healthy controls.	Skórkowska-Telichowska et al., 2009, Clin Invest Med [61].
Claudicant PAD	26/28	Apoptosis detection (by TUNEL Assay); Caspase activity measurement (by caspase Assay)	The fraction of TUNEL-positive nuclei was greater in PAD patients compared with controls (1.53% ± 0.96 for controls *versus* 3.83% ± 2.6 for PAD, *p* < 0.001). Caspase-3 activity was increased in PAD group compared with control group (0.22 ± 0.05 units mg^−1^ soluble protein *versus* 0.39 ± 0.09 for PAD, *p* < 0.001).	Mitchell et al., 2007, Vasc Med [59].

PAD: peripheral arterial disease; PCR: polymerase chain reaction.
